# Towards a Brazilian dementia plan? Lessons to be learned from Europe

**DOI:** 10.1590/S1980-5764-2016DN1002002

**Published:** 2016

**Authors:** Knut Engedal, Jerson Laks

**Affiliations:** 1 Professor, MD, PhD. Norwegian Advisory Unit for Aging and Health, Department of Geriatrics, Oslo University Hospital and Vestfold County Hospital, Norway.; 2 Associate Professor, Faculdade de Ciências Médicas, UERJ, MD, PhD. Institute of Psychiatry, Federal University of Rio de Janeiro, Programa de Pós-Graduação em Biomedicina Translacional - Biotrans, Universidade Unigranrio, Brazil.

**Keywords:** dementia, dementia plan, dementia strategy, diagnosis, dementia care

## Abstract

Dementia is a global socio-medical problem. The steepest increase in prevalence occurs in Latin-America and Asia. European governments have implemented dementia plans to improve care. We describe common goals of European dementia plans and discuss the Brazilian situation. Sixteen European countries have governmental dementia plans, another four are set to launch them. These plans have some common goals: to raise general awareness on dementia and reduce stigma, to establish more diagnostic centers and increase the number of people with correct diagnoses, to provide integrated care that improves quality of care and quality of life, and to promote educational programs for family and professional carers. European dementia plans have contributed toward raising awareness about dementia. More reference centers for diagnostic evaluations have been established and successful educational programs have been run. Integrated care is still a challenge in most countries. Brazil needs a plan. Facilitators and barriers for implementation should be identified by studying the European plans.

## RESUMO

Demência é uma problema medico-social mundial. O maior aumento da prevalência ocorre na América Latina e Ásia. Governos europeus implementaram planos para demência para melhorar cuidados. Nós descrevemos pontos comuns dos planos de demência europeus e discutimos a situação brasileira. Dezesseis países europeus têm planos governamentais de demência, outros quatro estão para lançar. Os planos têm objetivos comuns: estimular conhecimento geral e reduzir estigma., , estabelecer mais centros para aumentar o número de diagnósticos corretos, ferecer cuidados integrais que melhorariam qualidade de cuidados e qualidade de vida e para promoção de programas educacionais para familiares e cuidadores profissionais. Planos de demência europeus têm contribuido para estimular conhecimento sobre demência, aumentaram centros de referência para avaliações diagnósticas de demência, e programas educacionais têm sido montados. O cuidado integrado é desafio na maioria dos países. O Brasil precisa de um plano. Barreiras e facilitadores para sua implementação poderiam ser identificados através do estudo dos planos europeus.

## INTRODUCTION

It is well known that dementia will pose one of the biggest socio-medical challenges worldwide in the years to come.^1^ The current prevalence of any dementia disorder is about 44 million, a figure set to rise to over 100 million, and possibly to 140 million, by 2050.^1^ The steepest increase in prevalence will be seen in Asia and South-America.^1^ ^,^ ^2^ Most persons affected will be old people, no longer in the work force. Health personnel, health administrative staff around the world and even politicians, such as the former president of France, Nicolas Sarkozy, and the prime minister of England, David Cameron, have addressed this issue. The World Health Organization has placed the dementia challenge on its agenda and many governments have either drafted plans or already launched and implemented dementia-specific plans in order to improve care for people with dementia and their family carers (http/www.alzheimer-europe.org). Only recently, the Pan American Health Organization (PAHO) issued a document signed by all the countries in Latin America, stating that all countries are mandated to build programs for dementia as public measures to deal with the growing dementia prevalence rates in the region (http://www.alz.co.uk/media/151002). In Brazil, debates on the issue have just started, and there is still a need for an integrated, all inclusive, public and private task force to present a plan.

The aim of this chronicle is to briefly describe the common features of various dementia plans in Europe and to discuss whether Brazil should draft a nationwide dementia plan in order to improve care for people with dementia in the country. The manuscript is a chronicle based on various European dementia strategies and plans that have been published in the English language. The focus was on reporting common features of the various plans.

## EUROPEAN COUNTRIES WITH DEMENTIA PLANS

The two terms dementia plan and dementia strategy are used interchangeably in Europe. They are all developed by governmental bodies, and are therefore political documents. Content from these political documents will be presented in this article. Expert consensus statements made by a relevant professional body or an Alzheimer's association, or national guidelines will not be included.

The first European plan was launched in France in 2001 (2001-2005) and was a funded plan that focused mainly on medical aspects, such as establishing a network of reference centers, memory clinics and day-care centers, especially designed for people with dementia. The second French plan (2004-2007) was a continuation of the first plan launched without funding.

Later on, in 2008, the then-president Nicolas Sarkozy launched the third French dementia plan (2008- 2012) with the statement, "we will fight dementia", mirroring US president Richard Nixon´s launch of a cancer plan in 1971 with the statement "we will fight cancer". A comprehensive plan funded with 1.6 billion euros was launched. It contained 44 specific goals, each of them described in detail and funded.^3^ ^,^ ^4^ Other European countries followed suit.^5^ ^-^ ^8^ By the end of 2015, the following European countries had launched governmental plans or strategies (in the order launched): Norway, Belgium, England, Scotland, Wales, Northern-Ireland, Denmark, Finland, the Netherlands, Luxembourg, Switzerland, Greece, Ireland, Italy and Malta. Draft plans exist in Slovenia, Bulgaria, Cyprus and Portugal. In most of the countries, the plans launched between 2007 and 2014 had a duration of four years. This period in Scotland was three years, in Belgium six and in Norway eight years. Like in France, a second plan has been launched in both Scotland (2013-2016) and Norway (2016-2020).

## AIMS OF THE VARIOUS PLANS

The various European plans differ greatly, but the main aims are very similar^3^ ^-^ ^8^ and include: i) to improve the general awareness of dementia so that every citizen has basic knowledge about dementia and about the challenges associated with the disease; ii) to reduce stigma; and iii) to make efforts to ensure that people with dementia receive a disease-specific dementia diagnosis early in the course of the disease. Some plans focus on early diagnosis and others on timely diagnosis, but the main focus is that people should have a proper diagnostic assessment and if dementia is present, the diagnosis should be disclosed to the patient and the family, provided the patient consents to this disclosure. Moreover, the various plans further aim to improve the quality of care for people at any stage of the disease and thereby improve quality of life for people with dementia and family carers. In addition, some country-specific plans describe the goal of establishing a system for post-diagnostic follow-up throughout the course of dementia, including nursing home care and end-of-life care. Further goals in most countries are to improve knowledge about dementia among health care workers and family carers, and the skills of social and health care providers toward improving the quality of care.

## ACTIONS TO ACHIEVE THE GOALS

In some countries, the plans describe in detail which actions should be taken to achieve the goals; e.g. in France 44 actions are defined, in England 17 actions and in Norway five actions.^3^ ^,^ ^6^ ^,^ ^7^ The plans of other countries, such as the Swiss and the Danish plans, do not contain any detailed actions, but leave this to the local authorities (5). In addition, there are plans where actions are not clearly defined, such as in Greece.

### Some examples of major actions - short version 

*Actions aimed at the public.* Campaigns on Television and in other public media channels to raise the general awareness and reduce stigma have been carried out in many European countries. The effects of such campaigns are difficult to evaluate, but the general impression is that the awareness of dementia as a challenging social-medical problem has been raised significantly in many European countries over the last five years. An open National telephone line for information and support has been established for instance in France and Norway. Access is simple and open to everybody. It has been deemed a huge success. Particularly in the UK, Ireland and the Netherlands, the promotion of a dementia-friendly society has been focused and many local authorities have declared themselves as dementia friendly by educating the general population of their municipality about dementia.

The idea is to make daily life for people with dementia easier, so they can go shopping on their own and use public services.

*Actions to improve care for patients.* All European countries with a dementia plan have focused on how to improve access to diagnostic assessment and have a timely diagnosis. This has been achieved by establishing more memory clinics and/or other reference centers, both in primary and specialist health care systems. Some countries have established special services for people with early onset of dementia, such as specialized memory clinics (France), or post-diagnostic follow-up programs in the UK. The most costly actions are those related to follow-up programs after diagnosis. Different programs have been suggested. Access to new and existing services differs across countries, depending on the various political systems. However, one common idea is that patients with dementia and the family should have "one single point of contact", so that they do not have to dedicate great effort in the search for help. This "single point of contact" has different names in Europe, such as link worker, dementia coordinator, dementia team, dementia manager, single point of contact and so one. This contact person(s) or offices are the ones that should help the patient and the families receive the best integrated care that the local authority of the country they live in can offer.

Establishing special care units in residential care have been a focus in some countries such as France and Norway, whereas other countries have focused on how people with dementia can be treated well in general hospitals (UK).

*Educational programs.* One issue of many plans has been how to educate family and professional caregivers to support them in providing better care for people with dementias. Educational programs are normally easy to set up, and in many countries these have been successful. In Norway it has, for instance, been possible to set up family carer schools and educational programs for health personnel in almost all local authorities of the country at a very low cost. Similar educational programs for health personnel have been set up in many countries.

*Research.* Speeding up research, both basic biological research and care research, has been a goal in nearly all plans.

## FUNDING AND DURATION ARE KEY FACTORS FOR SUCCESS

Several factors are of importance for successful implementation of the dementia plans. The proposed actions should be acceptable for patients and carers while actions should be cheap or affordable or reimbursed (economic barriers). In countries such as France, where each action has been funded, it has been shown (unsurprisingly) that implementation is much easier compared to implementation of actions with low or no funding. In France, each of the 44 actions is funded specifically. Similarly, in the Norwegian plan some, but not all, actions are funded, albeit with a smaller amount of money than in the French plan. The actions with funding have been successful implemented (carer schools and educational programs for health personnel). Also, in the Netherlands, Ireland and Wales, there is fixed funding. In the Netherlands, this is in partnership between public and private agencies, whereas in England the actions that work well are funded. Greece and Switzerland are examples of countries where no extra funding is given to implement the national dementia plans. In some other countries such as Denmark, the funding to implement the plan is at a "symbolic" level.

Another aspect that facilitates implementation is the duration of the plan. In Norway, where the dementia plan had a duration of eight years, successful implementation of the carer school and the educational program for professional carers, was evident only three to four years after the dementia plan was launched.

 In the countries that had plans with well-defined goals, actions and funding, the implementation of actions was achieved better than in those countries where goals, actions and funding were loosely described in the plans.^4^ ^,^ ^9^ ^-^ ^13^

## DOES BRAZIL NEED A PLAN?

Brazil is the largest country in South America, with economic and social inequalities among regions, mainly between the North-northeast and South-southeast. As most epidemiological studies of dementia have been carried out in the Southeastern region, more data are needed from the regions not yet studied in order to develop a plan which can address particular needs of the population living in these regions. According to the available figures, there are an estimated 1.6 million older persons with dementia in Brazil.^14^ ^,^ ^15^ Also, a recent study which took place in a primary care program of a city in São Paulo state has shown that 77% of people with dementia had not been diagnosed.^16^ If generalized to Brazil as a whole, this would mean that about 1.2 million out of the 1.6 million people estimated with dementia are not diagnosed as such. These figures pose major challenges in the task of raising awareness and training health teams to diagnose and treat people with dementia.^16^ The low average educational level of the population in Brazil is also another important issue which has to be dealt with when developing measures to tackle the challenge in the plans. Education is a known risk factor for dementia and also poses many problems in diagnosing and treating dementia.^14^ ^,^ ^15^

Taking into consideration the experience of all the countries which have dementia plans, a national dementia plan should have clear, step-by-step goals, constantly discussed and checked against outcomes. Many government programs are already in place with regard to the general health and prevention of diseases in the elderly. For instance, there is a major program on hypertension and diabetes and a focus on family care professionals to deal with various problems.^17^ Training and improving knowledge and awareness on dementia among these professionals should be a main target of a new nationwide dementia plan that should have a duration of at least five years. Using the media to raise awareness may not be so expensive, and also the training of social and health care providers should be affordable. However, it should be noted that no national plan can be successful without full commitment of local, state, and national governments to accomplish it. Likewise, it is critical to the outcome of the program that all stakeholders and institutions linked to the health or social sector with a focus on older persons take part in the effort to map out a plan and take actions which are specifically designed for them. The knowledge obtained from the successful programs in Europe can help guide an initiative which is urgently needed in Brazil.

## COMMENTS

Some, but not all, of the plans have been evaluated and the main question in the evaluations has been whether the plan has led to better care for people with dementia. There is always a long way to go from planning an action to its implementation, and thereafter the question arises as to whether the action has made a difference for people with dementia and their families. 

The general awareness on dementia has definitely been raised in Europe over the last five to ten years. Whether this is the result of campaigns on TV, in newspapers and through other media channels is difficult to tell, but these have definitely contributed. Further, access to diagnostic assessment and a timely diagnosis has improved in most countries. Post diagnostic follow-up and establishing a system for provision of a chain of measures throughout the course of dementia for each patient has been successful only to some extent. Not surprisingly, new and better actions to improve care for people with dementia depend, among others, on the costs associated with care and whether funding exists to pay for the care. Further, major changes over a plan period of three to four years cannot be expected if specific actions are not funded. For example, the success of the Norwegian plan is probably a result of both the funding for specific actions and of a longer duration of the dementia plan.

What are the first steps to be taken in Brazil to design a national dementia plan? As shown above, dementia is still underdiagnosed to a great extent in Brazil. Developing programs to improve awareness in the public health system, mainly at the family health program level, by establishing training and education of the teams and by setting up dementia teams, could help tackle the problem, while also taking into consideration the inequalities and different cultural characteristics among the sociopolitical regions of Brazil. The dementia teams would be in charge of aiding diagnosis and providing advice for better care. Another important step would be to raise awareness at the political level, and to make every effort to try and raise funding, with the already well-established programs for hypertension and diabetes. One of the main barriers to the development of a dementia plan in Brazil seems to be the lack of a common policy to tackle the issue. Society at large, politicians, health care authorities, interested health societies, and third parties should get together and design a common way forward in dealing with the problem. Clearly, it is key to the success of a dementia plan that sufficient funding is secured for each level and step of the plan. As this is the most important barrier to success, it is fundamental that the plan be carefully designed and the appropriate funding carefully allotted, after relevant studies have taken place. Also, a constant check of the outcomes is needed.

## CONCLUSIONS

In Europe, an increasing number of countries have launched dementia plans aimed at improving care for people with dementia and thereby improving the quality of life for those with dementia disorders. Having evaluated the plans, the authors believe that without specific funding it is difficult to implement new services specially designed for people with dementia. Important actions include to raise general awareness about dementia, reducing stigma, to improve access to the health care system for a timely diagnosis, and to provide post-diagnosis support with a chain of measures tailored to each patient and their family.^4^ ^,^ ^9^ ^-^ ^13^ In Brazil, where an estimated 1.6 million people have dementia, only around one in four has been assessed and diagnosed. A nationwide dementia plan could both increase the number of people being diagnosed, but above all would improve the quality of care and quality of life for people with dementia and their families. Brazil can learn from the European plans which success factors and barriers are of importance, and how to implement new and better services for people with dementia.
